# Improving accuracy of cell and chromophore concentration measurements using optical density

**DOI:** 10.1186/2046-1682-6-4

**Published:** 2013-04-22

**Authors:** John A Myers, Brandon S Curtis, Wayne R Curtis

**Affiliations:** 1 Department of Chemical Engineering, Fenske Laboratory, The Pennsylvania State University, University Park, 16802, PA

**Keywords:** Optical density, Light scattering, Algae, Conversion factors, Dry weight, Flow cell

## Abstract

**Background:**

UV–vis spectrophotometric optical density (OD) is the most commonly-used technique for estimating chromophore formation and cell concentration in liquid culture. OD wavelength is often chosen with little thought given to its effect on the quality of the measurement. Analysis of the contributions of absorption and scattering to the measured optical density provides a basis for understanding variability among spectrophotometers and enables a quantitative evaluation of the applicability of the Beer-Lambert law. This provides a rational approach for improving the accuracy of OD measurements used as a proxy for direct dry weight (DW), cell count, and pigment levels.

**Results:**

For pigmented organisms, the choice of OD wavelength presents a tradeoff between the robustness and the sensitivity of the measurement. The OD at a robust wavelength is primarily the result of light scattering and does not vary with culture conditions; whereas, the OD at a sensitive wavelength is additionally dependent on light absorption by the organism’s pigments. Suitably robust and sensitive wavelengths are identified for a wide range of organisms by comparing their spectra to the true absorption spectra of dyes. The relative scattering contribution can be reduced either by measurement at higher OD, or by the addition of bovine serum albumin. Reduction of scattering or correlation with off-peak light attenuation provides for more accurate assessment of chromophore levels within cells. Conversion factors between DW, OD, and colony-forming unit density are tabulated for 17 diverse organisms to illustrate the scope of variability of these correlations. Finally, an inexpensive short pathlength LED-based flow cell is demonstrated for the online monitoring of growth in a bioreactor at culture concentrations greater than 5 grams dry weight per liter which would otherwise require off-line dilutions to obtain non-saturated OD measurements.

**Conclusions:**

OD is most accurate as a time-saving proxy measurement for biomass concentration when light attenuation is dominated by scattering. However, the applicability of OD-based correlations is highly dependent on the measurement specifications (spectrophotometer model and wavelength) and culture conditions (media type; growth stage; culture stress; cell/colony geometry; presence and concentration of secreted compounds). These variations highlight the importance of treating literature conversion factors as rough approximations as opposed to concrete constants. There is an opportunity to optimize measurements of cell pigment levels by considering scattering and absorption-dependent wavelengths of the OD spectrum.

## Background

### The Use of optical density

Optical density (OD) is used as a rapid proxy measurement of suspended biomass concentration. In fact, OD measurements are the most common measurement used in microbiology laboratories to assess microbial growth. It is used both qualitatively as the turbidity of a culture and quantitatively as a measure of the intensity of light transmitted along a path through the culture of known pathlength. While the visually-assessed turbidity of a culture is only a rough estimate of the cell concentration, it is useful in the context of prior experience culturing the organism. For example, OD is routinely used to identify the stage of growth for culture induction, inoculation and harvest. It is also presumed that OD correlates directly with biomass so that cell concentration can be monitored without having to conduct tedious procedures for measuring the cell dry weight or concentration of cells by hemocytometry or plating for colony forming units (CFU/mL). OD can also be used to estimate levels of pigments within cells when the chosen wavelenghth corresponds to the chromophore absorption. An objective of the analysis presented here is to promote the understanding of the effective use of OD as a proxy for monitoring biomass and cell pigmentation.

### The Beer-Lambert Law

The applicability of OD is largely discussed in terms of the Beer-Lambert law which relates the concentration of a sample to the attenuation of light as it passes through the sample.

(Eq.1) $$\mathrm{OD}=-\log\;\left[\frac{I}{I_{0}}\right]=\epsilon \;*\;c\;*\;l$$

In this equation, “I_0_” is the incident intensity of light, “I” is the intensity of light transmitted through the sample and measured by the photo sensor, “ϵ” is the attenuation coefficient, “c” is the concentration of the sample and “*l*” is the pathlength [1]. The attenuation coefficient (extinction cross-section) characterizes how strongly a suspension attenuates light. While most spectrophotometers have been standardized to a 1 cm path length, exceptions such as the 2.5 cm path length of the Klett-Summerson spectrophotometer do exist (Klett units). For applications in which the attenuation coefficient and the pathlength are constant, the Beer-Lambert law can be used to proportionately correlate biomass concentration ([X], g/L) with optical density (OD, at λ_i_/ 1 cm path length).

(Eq.2) $$\left[X\right]=m\cdot \mathrm{OD}$$

### Light scattering and absorbance

Since the scattering and absorption of light are the basis of using OD to measure biomass concentration and pigment levels, it is important to understand the underlying mechanisms. When light passes through a mixture, attenuation of the light intensity occurs by two mechanisms: in a homogeneous, single-phase solution, only absorbance contributes significantly to attenuation; in suspensions containing mixtures of two or more phases, light scattering due to difference in refractive index and the shape of this index mismatch also contribute to light attenuation [2]. The Beer-Lambert law only applies rigorously to single-phase solutions; however, certain assumptions and approximations allow the Beer-Lambert law to be extended to systems that exhibit significant scattering. As a mode of attenuation, absorbance specifically refers to light that is absorbed by the sample and subsequently converted to a different form of energy as opposed to being reemitted as light. Similarly, light that is attenuated by scattering must also initially be absorbed by the particle. “Scattering” then occurs when the dipoles induced by energy absorption reemit light in a different direction [3]. Depending on the configuration of the spectrophotometer and the angle of scattered light, this redirection can prevent the light from being detected by the photosensor. An additional complication to attenuation is that absorbance and scattering are not mutually exclusive. The ideal independent modes of attenuation result when the initially absorbed light is either exclusively converted to another form of energy or when it is reemitted at the same frequency (elastic scattering). However, inelastic scattering can occur when light is attenuated simultaneously due to absorbance and scattering in a singular event. In this case, some energy is retained by the particle before the light is reemitted at a lower frequency [3]. Since scattering takes place at all wavelengths, and absorption favors pigment-specific wavelengths, there is an opportunity to improve accuracy of the wavelength used for these OD measurement applications.

### Applicability of the Beer-Lambert Law to absorbing and scattering samples

**Figure 1:**
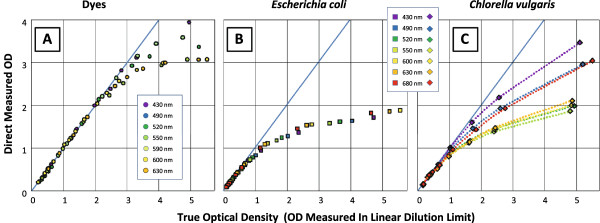
**The applicability of the Beer Lambert law for different types of samples.** The deviation from the linear Beer Lambert behavior is demonstrated at multiple wavelengths for samples of: **A)** various dyes, **B)***E. coli* cultures, and **C)***Chlorella vulgaris* algae cultures.

The inherent differences in particle absorbance and scattering affect the applicable range of the Beer-Lambert Law. Even though the specifications and sensitivity of the spectrophotometer dictate the maximum range of OD measurements, the relative contribution of scattering and absorbance attenuates the useful measurement range for a given sample. As seen in Figure 1, dyes, which predominantly attenuate light through absorbance, display the linearity of the Beer-Lambert Law to OD’s of around 2.5 on this particular spectrophotometer. Noting that this spectrophotometer is capable of a maximum absorbance of 4.0, the deviation of culminated transmittance from linearity is occurring due to the photosensor’s sensitivity. Since OD is based on a log scale, an OD of 2.5, corresponds to only 0.32% of the initial light reaching the photosensor. On the other hand, for an *E. coli* sample which has a significant light attenuation due to scattering, deviation from linearity occurs at a much lower optical density (Figure 1B, OD≈0.8). As the cell culture density increases, the number of scattering events per photon increases which adds complexity to the redirection of light in the spectrophotometer; a photon initially scattered away from the photosensor may be rescattered towards the photosensor [4]. This occurrence of multiple scattering events highlights the importance of measuring the OD at concentrations where the distance between particles is much greater than the size of the particles such that the number of scattering events per photon is kept to a minimum [3].

Additionally, since *E. coli* does not have significant pigmentation and associated absorbance peaks, the relationship between the amount of absorbance and scattering is relatively constant across the spectrum. As a result, a similar deviation is observed at different wavelengths. In contrast, for *Chlorella vulgaris* (an algae species), the deviation from linearity is wavelength-dependent due to the presence of photosynthetic pigments which increase the absorbance at certain wavelengths including the regions around 440-nm and 680-nm. Due to a substantial absorbance component, the deviation from linearity is delayed until higher ODs as compared to wavelengths where scattering dominates (Figure 1C). These observations illustrate the importance of understanding the contributions of scattering and absorption towards choosing measurement wavelengths that improve accuracy of OD correlations for biomass and chromophores.

### OD measurements will vary among spectrophotometers

**Figure 2:**
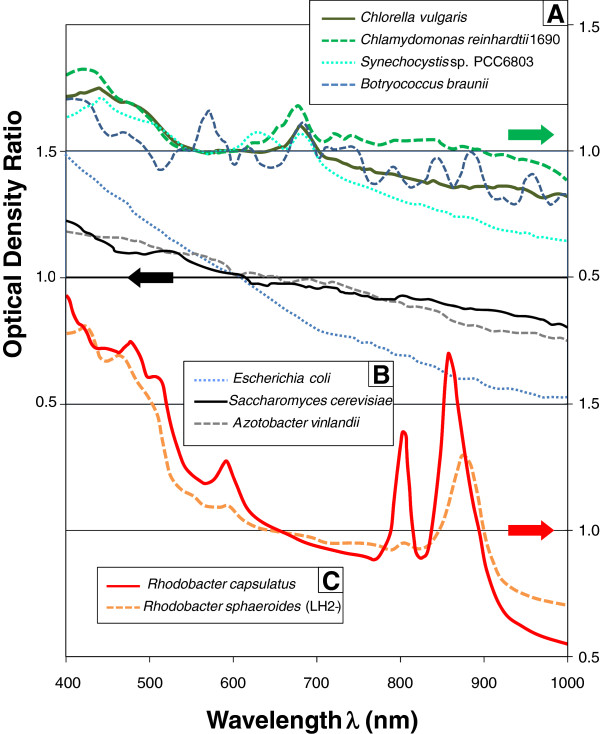
**Schematic representation of critical spectral elements for OD measurement.** The absorption spectrum of the sample attenuates a specific bandwidth of the light coming from the lamp spectral output and then quantified by the photo-sensor including its spectral sensitivity and ‘acceptance angle’ (red) for measuring photons.

Spectrophotometers vary substantially in the complexity with which they achieve measurements of light attenuation. Important elements of spectrophotometery are shown schematically in Figure 2. At a given wavelength the attenuation of light can be viewed as the difference of what is emitted and sensed (α → γ) with the ‘blank’, relative to what remains when the sample blocks photons (β → γ). Complexity arises because the photon emission spectrum varies tremendously for different light sources. Further, the specific wavelength is actually a bandwidth about that ‘λ’ because of the need to compromise between narrow bandwidth (with less intensity) and broader bandwidth (with greater intensity). The bandwidth tends to flatten the effective emission spectrum (indicated by dashed line in Figure 2) and resulting measured absorption spectrum. The photosensor will also have a wavelength-dependent sensitivity that defines an ‘acceptance angle’ that interacts with photons that impact at an angle off normal – which is particularly important for light scattering. Since OD measurements are dependent upon the photons available for assessing attenuation, the spectrophotometer-dependent characteristics of Figure 2 can all impact the accuracy of measurements within the wavelength-dependent OD spectrum.

**Figure 3:**
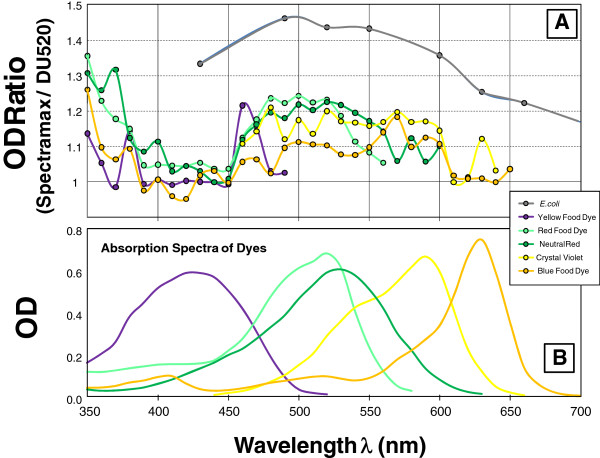
**Depiction of spectrophotometer differences for absorbing and scattering samples. A)** The ratio of the OD of different dyes measured on two spectrophotometers (the Molecular Devices Spectra Max 384 over the Beckman DU520) is plotted against wavelength to demonstrate the variability of spectrophotometers that is observed even for simple absorbing dye samples. An *E. coli* culture is included in the figure to illustrate how a predominantly scattering sample impacts the observed difference between these spectrophotometers. **B)** Absorption spectra of the dyes used.

In undertaking this study, the intention was not to evaluate spectrophotometer performance, but instead to gain an appreciation for the magnitude of the difference that would be encountered in using different spectrophotometers being used in the lab, as well as robustness of values of conversion factors for OD to biomass that are reported in the literature and used in productivity calculations. A comparison of dyes and *E. coli* were made to represent predominantly absorption and predominantly scattering (Figure 3A). The spectrum of the various dyes is shown for reference (Figure 3B). All of the measurements were taken in the linear range, and ratios of absorbance for the different spectrophotometers were calculated only when the absolute absorbance was greater than 0.05 (5% of full scale). A unit ratio on Figure 3A corresponds to identical measurements of OD for a given sample on the two different spectrophotometers. The observed ratio indicates that differences as large as 25% can be observed, even for a well-behaved dye solution. This variation between these two spectrophotometers depends on both the wavelength and the dye solution being measured. Figure 3A also presents the wavelength-dependent variation in OD ratio for *E. coli* measured on these two spectrophotometers. This variation reaches more than 40% and is consistently larger than observed for the dye solutions. This indicates that at least for these two spectrophotometers, there is a very pronounced difference that results from the scattering of light that occurs for the biomass particles in suspension.

**Figure 4:**
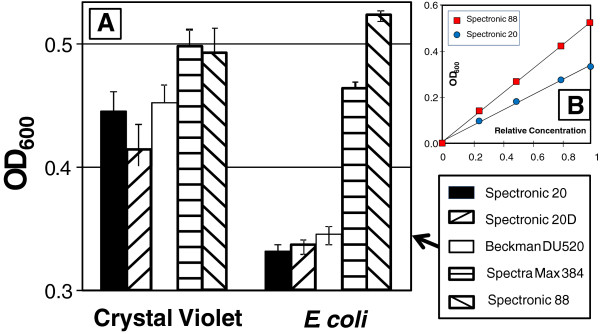
**Comparison of dye and *E. coli *OD measured on 5 different spectrophotometers. A)** Measurements of the same crystal violet solution (λ_max_ ~ 600 nm) and *E. coli* culture measured on 5 different spectrophotometers at well below saturating absorbance concentrations. **B)** Serial dilution of the *E. coli* sample on the two most disparate spectrophotometers to demonstrate linear correlation of OD with concentration.

A further comparison of 5 different spectrophotometers is summarized in Figure 4. The same dye solution of crystal violet gave significant differences for the different spectrophotometers, but displayed a variation that is less than 20%. Differences in the bulb output spectrum play a role in this inconsistency. In addition, the bandwidth will contribute variation which will become more important when the rate of change of slope for the emission or absorption spectrum becomes large in the immediate vicinity of the wavelength of interest. The *E. coli* samples displayed variation that was greater than 30% between the highest and lowest readings of the same sample (Figure 4A), suggesting a larger variation between spectrophotometers that results from light scattering. While these observations might lead one to question the general accuracy of optical density measurements, it is important to recognize that in all cases (both dyes and *E. coli* suspensions) the dilution profile on a specific spectrophotometer displayed nearly perfect linearity as indicated in the inset (Figure 4B) that shows this linear correlation for *E. coli* on the two most disparate spectrophotometers. This indicates that as long as measurements are taken on the same spectrophotometer, the resulting correlation with biomass concentrations can be very reproducible.

The preceding background analysis sets the stage for an assessment of the use of OD as a proxy for biomass measurements in a broad range of microorganisms. Focusing on the absorption contribution suggests methods for improving measurement of chromophore levels in pigmented cells as well.

### Scope

Certain considerations intended to optimize the utility and consistency of OD as a proxy measurement for cell biomass concentration are presented. This discussion focuses on the choice of blanking media and the selection of a wavelength of light. Manipulation of the contributions of scattering and absorption provide insight into interpreting OD measurements. With these considerations in mind, the use of OD to correlate to both dry weight, cell count and pigment content is discussed in terms of its useful but limited scope. Finally, a short path length flow cell is discussed in terms of its ability to be used for continuous bioreactor monitoring.

## Methods

The five spectrophotometers compared in this work were a Bausch and Lomb Spectronic 20, a Milton Roy Company Spectronic 20D, a Bausch & Lomb Spectronic 88, a Beckman DU 520, and a Molecular Devices Spectra Max 384 Plus.

**Figure 5:**
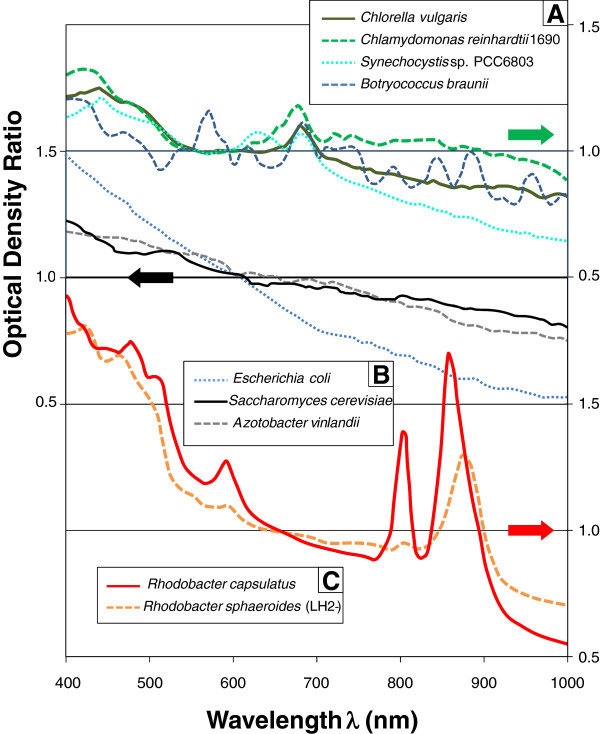
**Demonstration of the variation in absorption spectrum among diverse microorganisms.** Absorption spectra of a wide variety of microorganisms, obtained on a Spectramax photometer measured at OD_R_ ~1 are grouped as follows: **A)** Spectra of green photosyntyetic cultures normalized to an OD_550_ of 1.0, **B)** Spectra of un-pigmented cultures normalized to an OD_600_ of 1.0, **C)** Spectra of red cultures were normalized to an OD_660_ of 1.0.

Absorption spectra of the cultures were obtained on actively growing late phase cultures with dilution to a density of about 0.2 at the nominally robust wavelength using the most sensitive spectrophotometer (Molecular Devices Spectra Max 384). In all cases except for the spectrum for *E. coli*, tap water was used for blanking and sample dilution. Since *E. coli* was grown on a complex media that was known to significantly contribute to the attenuation at the low end of the spectrum, dilution and blanking was conducted with the culture supernatant. The absorption spectra of the two *Rhodobacter* species were taken at higher densities (OD_660_ = 1.85 for *sphaeroides* and OD_660_= 1.41 for *capsulatus*) in order to accentuate the absorbance peaks. It is worth noting that the relationships between the ODs at the different wavelengths are therefore not accurate for these spectra (Figure 5) due to the measurement in the attenuated high cell concentration range.

Dry weights were generally obtained by centrifuging a 5–10 mL sample, rinsing with tap water into a pre-tarred epitube. Dry weight of the final cell pellet was determined by difference after freeze-drying. CFU measurements were typically obtained by spreading 100-mL of culture on 9-cm plates to obtain 100–400 colonies on an appropriate growth medium. Cultures were diluted with 1/10x fresh media in 25-mm flat bottom culture tubes and spread with a 25-mm triangular end bar spreader.

Culture Sources and Media (additional details in acknowledgements): *Azotobacter vinelandii* was obtained from the ATCC, American Type Culture Collection (http://www.atcc.org, ATCC 478, grown on AIC medium), *Bacillus thurengensis* ssp israelensis was obtained from the Bacillus Genetic Stock Center (http://www.bgsc.org, 4Q5, LB medium, 37C). *Chlamydomonas reinhardtii* was obtained from the Clamydomonas Resource Center (http://chlamycollection.org/, cc-1680 w.t., cc-503 cell wall mutant, TAP medium, 25C), *Chlorella vulgaris* was obtained from the UTEX culture Collection of Algae (http://web.biosci.utexas.edu/utex/, UTEX 2714, TAP medium), *Escherichia coli* K91 for the 5-L fermentation run was obtained from CGSC, the *E. coli* Genetic Stock Center (cgsc.biology.yale.edu/, minimal modified M9 medium + fed batch 28% NH_4_OH, 555 g glucose /L, 37C), *Nanochloropsis oculata*, originated from CCMP, National Center for Culture of Marine Pyhytoplankton (ccmp.bigelow.org, CCMP 525 , f/2 medium, 25C), *Saccharomyces cerevisiae* and *Candida molischiana* were obtained from the USDA NRRL ARS Culture Collection (nrrl.ncaur.usda.gov/, S.c. NRRL #Y-562, C.m. NRRL #Y-2237, YMP medium + cellobiose, 30C). Additional details of culture media are provided at the Curtis Lab web site: http://www.curtisLab.org Optical densities, dry weight and CFU were measured on mid-to-late logarithmic phase batch cultures unless otherwise noted. Dyes were obtained from the following sources: crystal violet (EM Science, 97%, C.I. 42555), red & blue food color dye (McCormick, Red #3 and #40, blue #1 with 0.1% propylparaban ), Neutral red (Fisher, C.I. 50040).

## Results and discussion

### Choice for blanking the spectrophotometer can be important

A basic consideration when measuring the OD of a culture is selecting a suitable blank. Depending on the nature of the work, there are three different blanking materials commonly utilized for biological samples: water, fresh growth media and the supernatant of the culture. When blanking with water, the problem arises that some of the measured attenuation will be the result of media constituents as opposed to the cells themselves. However, most defined media demonstrate very little attenuation in comparison to water and this problem is minimal. On the other hand complex media containing cell or protein hyrolysate contribute to absorbance in the 350 to 550 nm range. If fresh growth media is used as a blank, the media contribution is included, but complications arise due to the media’s fluctuating contribution to the OD as the cells begin to consume nutrients. Arguably the most accurate approach to correlating suspended biomass is to use the culture supernatant as a blank, so that the measured light attenuation results from the cells and the multiple reflection profile of light within the cuvette approaches that of the sample. If the sample needs to be diluted prior to OD measurements, the more rapid deviation from linearity due to scattering (Figure 1B) should be kept in mind and dilution to below OD≈0.5 is often recommended. If a medium other than supernatant is used for dilution, this can be taken into account and corrected for.

### Selection of wavelength is critical for both pigmented and unpigmented cells

The choice of wavelength is an important consideration for monitoring and correlating optical density with biomass concentration [5]. Variation in pigment profile among organisms yields great diversity in absorption spectra, especially among highly-pigmented strains (Figure 5). A diversity of organism appearance is illustrated in the ‘microbial collage’ of the supplemental figure (Additional file 1: Figure S1). The absorption profiles of the unpigmented bacteria display largely monotonically decreasing absorption as wavelength increases. *E. coli* culture cell concentrations have been reported in the literature at various wavelengths including 420, 460, 590, 600, 650 and 660-nm [5-9]. It might be presumed that since *E. coli* is not pigmented, the choice of wavelength does not matter. However, as can be seen from Figure 5, that among the non-pigmented bacteria, *E. coli* displayed the largest variation, steadily declining from 400 to 1000-nm. This decline results as the Rayleigh regime is approached where scattering follows an inverse fourth power with wavelength. This means that sensitivity will decline as well as an accompanying increase in dry weight per OD as higher wavelengths are used. The profile is observed to be very consistent for a given spectrophotometer which means that a correction factor (δ) can be applied to convert between different wavelengths.

(Eq.3) $$\begin{array}{l} \left[X\right]=m_{1}\cdot OD_{1}=m_{2}\cdot OD_{2}\\ \;=\left(\delta \cdot m_{2}\right)\cdot OD_{1}\Rightarrow \;\delta =\frac{m_{1}}{m_{2}}=\frac{OD_{2}}{OD_{1}} \end{array}$$

Extrapolating wavelength-dependent behavior obtained with different spectrophotometers can lead to erroneous results. Comparing a typical algae reference wavelength of 550-nm to an alternative wavelength of 730-nm for cyanobacterium *Synechocystis* sp. PCC6803 resulted in δ>1 for one spectrophotometer but δ<1 for another (data not shown). This is a reminder that the lamp and photosensor play a role in the resulting absorption spectra as depicted in Figure 2.

**Figure 6:**
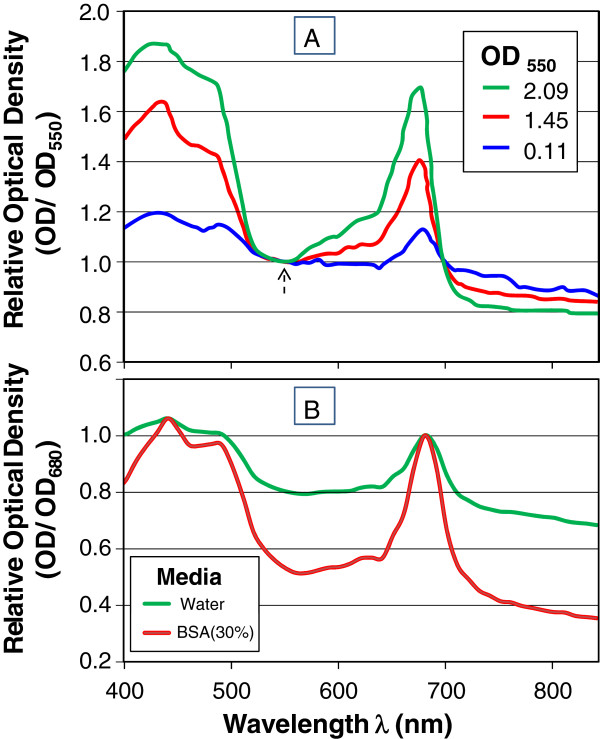
**Factors which strongly effect relative contribution of scattering and absorption. A)** Absorption spectra of *Chlorella vulgaris* measured at three undiluted concentrations and then normalized to the robust optical density at 550 nm. **B)***Chlorella vulgaris* spectra measured when resuspended in tap water or 30% bovine serum albumin (BSA) to reduce the scattering component of light attenuation.

The peaks in the absorption spectra correspond to pigments (such as chlorophyll in algae; Figure 5A), and light harvesting antenna in purple photosynthetic bacteria *Rhodobacter* (Figure 5C). The greater opportunity for observing absorption peaks for lower energy, higher wavelength light is apparent in the gradual decline in the attenuance spectrum at higher wavelengths that is particularly evident for the non-pigmented cultures (Figure 5B). The influence of the mechanism of light attenuation on OD at different wavelengths is clarified in Figure 6A. The peak absorbances are shown to be accentuated for spectral measurements carried out at higher cell concentrations. While this may seem counter-intuitive based on the saturation of absorbance measurements that occur at higher density, it must be remembered that the actual measurement is light attenuation. As the concentration of cells between the light source and sensor is increased, the scattering component of the total light attenuation falls off more quickly than the absorption component (Figure 1). As a result of multiple scattering events, the extinction of light due to scattering is attenuated relative to absorption and the relative peak heights are higher (Figure 6A). To further explore the relative contribution of scattering and absorption within an ‘extinction spectra’, cells were resuspended in solutions of bovine serum albumin (BSA). By bringing the refractive index of the solution closer to that of the cells, the scattering component can be dramatically reduced as shown in Figure 6B, where the chlorophyll peak of this chlorella culture is greatly accentuated when measured in a BSA solution. A nearly proportional drop in the non-pigmented (scattering) components of the spectrum is observed for the light-harvesting complexes of *Rhodobacter capsulatus* for a range of BSA from 0-40% (Additional file 2: Figure S2). This technique shows the potential value of reducing scattering to observe pigmentation for example in screening based on absorbance or color (e.g. carotinoids, fluorescent proteins). This approach to refractive index matching can reduce scattering which has been shown to dominate light attenuation in algae cultures [10].

There are two competing technical considerations when a wavelength for OD measurement is selected: **sensitivity** and **robustness**. At a robust wavelength, the relationship between OD and biomass is largely independent of the growth conditions (OD_R_ = OD_Robust_). The consistency of such robust correlations stems from the fact that at these wavelengths, scattering of light is relatively unaffected by the major pigments in the cell. A robust wavelength is chosen from a region of the spectrum that is away from the absorption peaks if they are present. In these regions, the mechanism of light attention is predominantly scattering which is relatively constant due to consistent cell biomass composition and associated refractive index. For an un-pigmented culture such as *E. coli*, virtually any wavelength could be used, but the OD @ 600-nm has become a common convention – in part due to the ease of generating and measuring this wavelength within the visible spectrum. Certain limitations of a robust wavelength’s consistent correlation to biomass are discussed in the “Correlations to Biomass and Cell Count” section.

**Figure 7:**
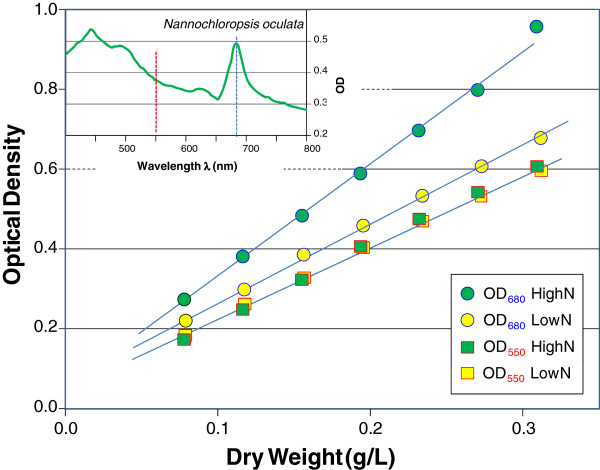
**OD measurements which avoid absorption peaks provide robust biomass correlation.** Correlation between biomass density and optical density for *Nannochloropsis* algae cultures grown on high and low nitrogen to alter the chlorophyll content. Measurements corresponding to chlorophyll absorbance (~680 nm, figure inset) display very different correlation as compared to the correlation at the robust wavelength (OD_R_) of 550 nm.

In contrast to the relatively constant biomass proxy correlation at robust wavelengths, the relationship between OD and biomass concentration at sensitive wavelengths can vary considerably with culture conditions that effect the level of absorbing species such as pigments within the cell. Because ‘pigments’ have large attenuation coefficients by definition, slight fluctuations in the concentration of a given pigment can result in significant changes to the relationship between a sensitive OD and biomass. For example, the light-harvesting proteins of *Rhodobacter* (Figure 5C) are completely absent under aerobic conditions and dramatically induced under anaerobic photo-heterotrophic conditions [11]. To demonstrate the consistency of a robust wavelength’s correlation to biomass and the variability of a sensitive wavelength’s correlation to biomass, a comparison was made between samples of *Nannochloropsis* alga grown in media with different nitrogen concentrations. Cultures grown in high concentrations of nitrogen developed a darker green color than cultures grown in low concentrations of nitrogen - which became yellow-green as the result of significantly reduced chlorophyll concentrations. Despite these differences in growth conditions, it was demonstrated that the correlations between dry weight and the robust OD at 550-nm were almost identical for the two cultures (0.492 g/L/OD_550_ for the high nitrogen algae versus 0.501 g/L/OD_550_ for the low nitrogen algae) (Figure 7). However, the relationship between the dry weight and the OD at 680-nm differed by 34% (0.325 g/L/OD_680_ for the high nitrogen algae versus 0.437 g/L/OD_680_ for the low nitrogen algae). The smaller ratio of the DW to OD for the green, high nitrogen algae is indicative of a higher concentration of chlorophyll, which absorbs light around 680 nm.

Despite the fact that the correlation between biomass and the OD at a sensitive wavelength is subject to change with conditions, for constant environments such as the sea, sensitive wavelengths, such as 680-nm, are the preferred wavelength to estimate phytoplankton levels. Using a sensitive wavelength such as the 680-nm absorption peak of chlorophyll, has the advantage of facilitating quantification of very low concentrations of phytoplankton. Additionally, because the chlorophyll peak stands out in contrast to other seawater constituents, algae content can be differentiated from simply cloudy water. The observation of a pigmentation-dependent correlation parameter (m_λ_), suggests that pigment levels can be quantified based on ratios between absorbing and non-absorbing wavelengths. To correlate cell pigment content with OD measurements requires correcting the OD measurement at the absorbent wavelength for its scattering component. This approach is explained in the supplemental material (Additional file 3: S3) where the exclusion dye trypan blue is quantitatively added to *E. coli* culture to separate the absorption and scattering components.

### Optical density provides biological conversion factors for biomass and cell count

There are additional considerations beyond ‘pigmentation’ that affect the ability to correlate optical density with biomass and cell concentrations. In general, anything which affects either light absorption or scattering is a potential candidate. As an example, we have observed the algae *Botryococcus braunii* to change in colony aggregate size between 0.09 to 0.34 mm for different continuous growth rates. This change in aggregate size is associated with a 2-fold change in the conversion factor at the robust wavelength (m_R_), reflecting this large proportional change in DW/OD_550_ as a result of the large changes in light scattering over this range. Additionally, some organisms tend to secrete materials such as polysaccharides under various conditions which can significantly alter the culture absorption spectrum. Because of these factors, it is often necessary to consider potential variations to a DW/OD correlation when the physiological growth conditions, {*z*_*i*_} change substantially. It may be possible to quantify that change with an additional parameter measurement as a function of those conditions, m{*z*_*i*_}:

(Eq.4) $$\left[X\right]=\left(m_{R}\left\{z_{i}\right\}\right)\cdot OD_{R}$$

Despite the limitations for use of OD, it remains a powerful index of microorganism growth as a result of simplicity and speed. The literature is full of growth and productivity information that is based on these measurements. As a result, biological conversion factors between the quantities of optical density (OD), cell density (gDW/L) and cell count (CFU/mL) are extremely useful. We have tabulated dozens of references to these conversion factors in the literature, but are reluctant to infer trends from this information due to the complexities of differences in spectrophotometers and methods of handling dry weight measurements and counting of cells. Recognizing the utility of a more consistent compilation, our laboratory undertook a survey of these biological conversion factors. This work included a diverse range of organisms (algae, bacteria, yeast) and range of growth conditions (heterotrophic, phototrophic and autotrophic). Our intention was to not simply compile average values, but report standard deviations that were representative of the various researchers that were responsible for the culture of this wide range of organisms (Table 1).

**Table 1:** Conversion factors between OD, dry weight and cell number

Species*	**OD**_**robust**_	**spec**	**gDW/OD**_**robust**_	**CFU/OD**_**robust **_**(x10**^**-8**^**)**
*Escherichia coli* (DH5α)	600	A	0.396 ± 0.011	7.94 ± 2.0
	600	B	0.515 ± 0.014	10.3 ± 2.6
*Agrobacterium tumafaciens* (EHA105)	600	A	0.409 ± 0.004	13.66 ± 7.06
	660	A	0.485 ± 0.004	16.19 ± 8.37
*Agrobacterium rhizogenes* (15834)	660	A	0.410 ± 0.011	7.89 ± 0.95
		A	0.509 ± 0.014	9.40 ± 1.13
*Azotobacter vinelandii*	600	A	1.920 ± 0.205	4.50 ± 1.91
*Bacillus thuringensis* (ssp. israelensis)##	600	A	0.335 ± 0.006	0.519 ± 0.183
	660	A	0.370 ±0 .007	0.573 ± 0.202
*Clostridium phytofermentans* (ISDgT)	600	A	1.64 ± 0.05	6.47 ± 1.34
	600	B	2.43 ± 0.07	9.58 ± 1.98
*Rhodobacter capsulatus* (SB1003)				
Heterotrophic (aerobic)	660	A	0.626 ± 0.048	244 ± 104
	660	B	0.652 ± 0.052	251 ± 199
	600	A	0.500 ± 0.036	223 ± 164
	600	B	0.548 ± 0.042	261 ± 193
Photoheterotrophic (anaerobic)	660	A	0.419 ± 0.031	13.9 ± 5.4
	660	B	0.499 ± 0.061	14.8 ± 6.1
Autotrophic (H_2_, O_2_, CO_2_)	660	A	0.563 ± 0.003	43.2 ± 5.6
	660	B	0.602 ± 0.003	45.5 ± 5.9
*Rhodobacter capsulatus* (SB1003)				
Heterotrophic (aerobic)	660	A	0.453 ± 0.144	367 ± 122
	660	B	0.515 ± 0.128	378 ± 126
	600	A	0.466 ± 0.074	341 ± 113
	600	B	0.491 ± 0.111	312 ± 104
Photoheterotrophic (anaerobic)	660	A	0.553 ± 0.004	159 ± 27
	660	B	0.606 ± 0.005	174 ± 29
	600	A	0.446 ± 0.004	128 ± 22
	600	B	0.548 ± 0.004	158 ± 27
*Ralstonia eutropha* (h16)				
Heterotrophic (aerobic)	600	A	0.464 ± 0.017	11.5 ± 2.2
	600	B	0.604 ± 0.022	13.3 ± 2.5
Autotrophic (H_2_, O_2_, CO_2_)	600	A	0.298 ± 0.007	16.7 ± 1.1
	600	B	0.383 ± 0.009	20.8 ± 1.4
*Saccharomyces cerevisiae* (Y-562)	600	A	0.644 ± 0.025	0.330 ± 0.083
	600	B	0.848 ± 0.033	0.430 ± 0.109
*Candida molishiana* (yeast)	600	A	0.436 ± 0.011	0.498 ± 0.104
*Synechocystis* sp. PCC 6803	550	B	0.63 ± 0.11	0.380 ± 0.046
	730	B	0.60 ± 0.10	0.478 ± 0.058
*Synechococcus* OH2	750	A	0.361 ± 0.010	-na-
*Synechococcus* sp. (Marine )	750	A	0.350 ± 0.020	1.05 ± 0.100
*Chlamydomonas reinhardtii* (1690)	550	B	0.530± 0.100	0.240 ± 0.028
*Chlorella vulgarus*	550	A	0.316 ± 0.007	0.161 ± 0.019
	550	B	0.430 ± 0.024	0.692 ± 0.077
*Botryoccoccus braunii* (race B)	550	B	2.15 ± 3	-na-

Spec A = spectramax; Spec B = Beckman DU.

## plasmid cured strain; $$ cell wall mutant; *organism characteristics readily accessed via microbewiki.

Optical density at various robust wavelengths (OD_R_) was measured in conjunction with measurements of dry weight concentration (gDW/L) and colony forming units (CFU) as measured by dilution plating. Growth media and conditions were those typically used for these various organisms.

Beyond providing a range of useful biological conversion factors (OD-DW-CFU), numerous surprising observations were obtained. The dry weight to robust OD ratio (gDW/OD_R_) ranged for the majority of organisms between 0.35 (*E. coli*) to 0.65 (yeast) with most algae, cyanobacteria and other pigmented bacteria such as *Rhodobacter* falling between this range. As noted above, the colony-forming algae *Botryococcus braunii* has an exceptionally high gDW/OD_R_ due to forward-scattering of light that occurs for large particles. *Clostridium phytofermentans* also had a surprisingly high dry weight per OD, that is apparently due to clumping or sporulation of this bacteria species (though not macroscopically apparent by visual observation). The aggregate of this data suggests that a value of 0.5 gDW/OD_R_ is a reasonable approximation for most cultures which is a bit surprising given the broad range of organisms and ranges of wavelengths used (550–750 nm). This relatively narrow range of dry weight correlation values (m_R_), results because of the general approach to use a wavelength measurement where light-scattering dominates over specific absorption as the mechanism of light attenuation.

A much more surprising result of this compilation is the conversion factor for colony forming units per robust OD (CFU/OD_R_) varied over a range of 2 orders of magnitude. Although CFU is inherently more difficult to obtain (and has a standard error that is closer to 25%) as compared to the dry weight measures (which usually showed less than 10% variation), this does not account for this tremendous range of measurements, and illustrates the very large difference in the number of cells present within the culture at unit optical density (OD_R_ =1). For bacteria, a value of 1 × 10^9^ CFU/OD is often cited. This value is close to our value of 8.0x10^8^ CFU/OD_600_ for *E. coli*, particularly since this is for the Spectramax spectrophotometer which tends to read on the higher side for optical density (Figure 4); the value for the more common Beckman spectrophotometer is equal to this nominal value. *Rhodobacter* species, particularly *capsulatus* displayed CFU/OD_660_ > 300 × 10^8^. For anaerobic photoheterotrophic growth condition. In contrast, the biocontrol agent *Bacillus thuringensis* had nearly 1000-fold less bacteria at 0.52x10^8^ CFU/OD_600_, which is 10-fold less bacteria than *E. coli* as this same robust wavelength (λ_R_) for non-pigmented cells.

While there is a tendency for the larger eukaryotic cells to have smaller CFU/OD, *Rhodobacter* displays a substantially higher CFU as comparable size *E. coli* (0.5 × 2 μm) at nearly the same dry weight when measured at the same wavelength. It is an interesting observation that light passes through a photosynthetic culture of *Rhodobacter* more easily than for example a soil-derived *B. thuringensis*. Noting that this is not at the absorption peak it still suggests a greater degree of ‘transparency’. It is noteworthy that this tendency persists for different growth conditions (hetero-, photo-, auto-trophic) which would be consistent with a physical characteristic of the cells since they are so dramatically different in pigmentation (Additional file 1: Figure S1). Interestingly, autotrophic *Ralstonia*, which does not utilize light energy, does not display a disproportionately high CFU/OD_R_. The diverse green ‘micro-algae’ and cyanobacteria are surprisingly consistent at about 5x10^7^ CFU/OD_R_ which is on par with the yeast species. The extreme outlier of *Botryococcus* illustrates the tendency of an aggregated culture to allow transmission of light and thereby represent a higher cell mass for a given optical density. The aggregate of this data along with the different wavelength and spectrophotometers provides an idea of how generally applicable this type of biological conversion factor can be relied upon to provide quantitative productivity calculations. The greatest accuracy will be obtained by establishing the biomass correlation with the specific spectrophotometer that will be used during monitoring. Estimates provided from different spectrophotometers would generally be within 25% although the error could be larger for specific spectrophotometer combinations. In many cases, the absolute biomass concentration is not as important as a relative comparison for experimental treatments. However, with the increased vision of microorganisms as biochemical production platforms for chemicals and fuels, there are associated calculations of productivity where 10% difference is very important.

### A short path length OD flow cell can be used for continuous online monitoring

**Figure 8:**
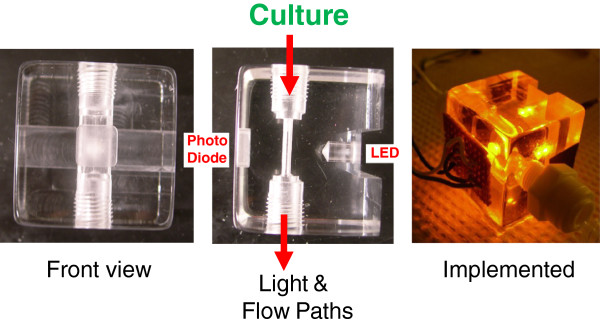
**A short path-length optical density flow cell for bioreactor monitoring.** A flow cell spectrophotometer machined from an acrylic block to accommodate an LED light source on one side of the culture flow slit, and a photodiode on the other. L.T.R.: Front view where the photodiode senses light; side view showing the LED position relative to the flow path; flow cell implemented for monitoring of *E. coli* culture.

One of the biggest limitations using OD is saturation (or more precisely lack of sensitivity of measurement as nearly all of the light becomes attenuated). Referring to Beer-Lambert law (equation 1) the alternative to diluting a sample or using reduced absorbance region of the spectrum is to reduce the light path length. To achieve this reduction in optical thickness, an OD flow cell was fabricated by machining a slit through an acrylic block (Figure 8). By reducing the path-length within the flow cell from the standard OD path-length of 1 cm to roughly 0.1, the applicable range of the device is significantly extended. Fabrication details, including a mechanical drawing are provided as supplemental material (Additional file 4: Figures S3A & B). An LED used as the light source has the advantage of being an inexpensive long-lasting light source with reasonably narrow bandwidth (although only adjustable by changing the LED). Light levels are sensed with a Burr-Brown photodiode OPT101P that is fitted into a window to hold the 8-DIP chip in place and biomass concentration is measured as the attenuation of voltage from the photodiode sensor. This design is particularly useful for a dedicated monitoring spectrophotometer that can be implemented for continuous monitoring of growth. Online OD monitoring not only reduces manual sampling, but also allows for automated bioprocess monitoring and control.

**Figure 9:**
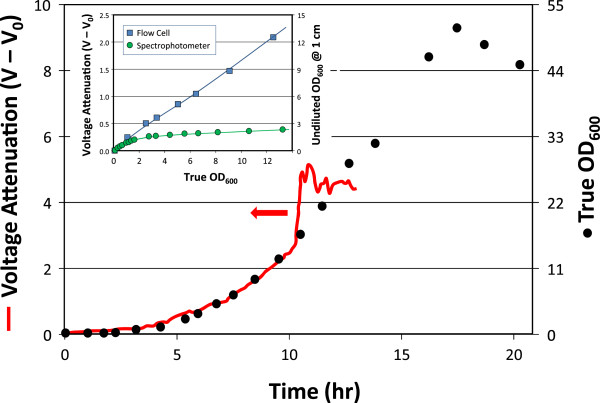
**Online implementation of the OD flow cell to monitor an *E. coli *fermentation.** Measurement of OD time course in a 5-L fed-batch *E. coli* fermentation using a peristaltic pump to pass culture through the flow cell in a recirculation loop. The OD flow cell was implemented with a 595 nm LED (Ligitek, LHY3833, 2700-mcd, 12^o^). The true optical density was measured offline after dilution to the non-saturating biomass concentrations on Beckman spectrophotometer. The inset is a plot of the non-diluted OD-biomass correlation measured in the flow cell as compared to the offline spectrophotometer.

The online monitoring of a fed-batch *E. coli* fermentation is shown in Figure 9. Growth was successfully monitored to an OD_600_ > 12. At this point, the density of the culture was sufficiently high that cells were sedimenting out within the flow line to and from the flow cell and the failure was not due to saturation of the flow cell measurement. As shown in the inset, undiluted off-line measurements had saturated long before OD_600_ = 1 was achieved, while online measurements were nearly liner for more than an order of magnitude higher optical density. Ongoing efforts are improving the geometry and flow pattern within the OD-cell, as well as greatly reducing the cost using ‘open source’ Arduino™ interfacing hardware. It is important to recognize that in this application of correlation of OD with biomass, it is not necessary to achieve the ideal linearity associated with equation 2. As long as OD does not saturate and become independent of cell concentration, a correlation can be established. This behavior is illustrated in Additional file 5: Figure S5 where *Chlorella* cultures at an OD up to 10 were run through the OD-cell. While curvature is clearly apparent, the goal of correlation biomass can still be accomplished by fitting an appropriate equation that describes saturation of the photo-sensor.

(Eq.5) $$\left[X\right]=\frac{K\cdot OD_{R}}{\left(OD_{\text{saturation}}-OD_{R}\right)}$$

Where ‘K’ is the cell concentration at half-saturation of the OD_saturation_ at which the measurement of optical density saturates and will not change at higher cell concentrations.

This approach to fitting optical density data rather than conducting dilution has been published as a brief research method where the non-linearity of the relationship between true cell concentration and OD can be captured from the serial dilution OD profile of a dense culture [12]. This approach of defining relative density is particularly useful for assessments of doubling time where absolute cell concentrations are not required. As long as measurements are conducted in the same spectrophotometer, at a fixed wavelength that remains robust throughout the growth time course, fitting is a valid alternative to dilution. The accuracy of the correlation will then become dependent upon the local derivative of the measurement (d[X]/dOD_R_), which tends to be accurate as long as measurements are well below saturation. This further illustrates the value of our short pathlength flow cell where mathematical calculations can be easily incorporated into software that will facilitate the calibration fitting and then output of the desired biomass concentration to monitor microorganism growth. Besides obvious limitations such as changing pigmentation, it should be kept in mind that total light attenuation is the sum of both scattering and absorption components. Since these components behave differently (Figure 1), and the sum of two saturation curves does not necessarily give a simple saturation behavior, alternative functional forms for Equation 5 may be required to provide an accurate correlation between optical density and biomass concentration.

## Conclusions

Optical density (OD) can be a very useful tool for correlating to biomass and determining relative pigment concentrations; however, it has certain limitations. When comparing conversion factors found in the literature, it should be acknowledged that there are potentially large differences between spectrophotometers, and such conversion factors are dependent upon wavelength and changes in culture conditions. None-the-less, accurate correlations and associated conversion factors can be developed when the concepts of light scattering and absorption are considered. If a correlation is desired for biomass, then a robust wavelength dependent primarily on scattering is preferred. If a correlation is desired for chromophores, a sensitive wavelength combined with manipulation of media refractive index can provide the most accurate measurement. Additionally, a short pathlength flow cell can provide accurate online measurements of optical density at densities that would otherwise require dilution to prevent ‘saturation’ of OD measurements that result when insufficient light reaches the photodetector. The search for sustainable production of bioproducts is resulting in rapid expansion of the diversity of organisms being considered as production platforms which requires quantitative assessments of biomass productivity. This work has been carried out to facilitate a greater understanding of the potential and limitations of optical density measurement to improve the accuracy and utility of those measurements.

## Abbreviations

α-γ: Photons which reach sensor with ‘blank’ present (Figure 2); β-γ: Photons which reach sensor when sample is present (Figure 2); δ: Ratio of biomass conversion factors at different wavelengths (Eq.3,S3.2,S3.3); ϵ: Attenuation coefficient (Eq.1); λ: Wavelength of light; c: Concentration of the sample (Eq.1); CFU: Colony forming units; gDW: Grams dry weight; I: Intensity of light transmitted through the sample and measured by the photo sensor (Eq.1); I0: Incident intensity of light (Eq.1); K: Parameter for non-linear ‘saturation’ dependent correlation of biomass to OD (Eq.5); l: Pathlength (Eq.1); m: Proportionality constant/conversion factor between OD and DW (Eq.2,3,4); OD: Optical density; P: Optical density contribution due to pigment absorption (Eq.S3.1); X: Biomass concentration, gDW/L; zi: Indicates a dependence on different growth conditions (Eq.4).

## Competing interests

The authors declare that they have no competing interests.

## Authors’ contributions

JAM carried out spectrophotometer model, wavelength, and dye comparisons. JAM and BSC coordinated and provided training for the overall culture effort, spectrophotometric and dry weight analysis. WRC fabricated the OD flow cell and implemented for the *E. coli* batch run. WRC also carried out the *Nannochloropsis* wavelength study. The manuscript was written and edited by JAM, BSC, and WRC. All authors read and approved the final manuscript.

## Authors’ information

WRC’s appointment at Penn State included a role to provide technical assistance to a bioprocessing scale up facility. This 3000sq.ft. facility included microbial and cell culture areas and assisted small and large biotechnology companies with scale up starting in the 1990’s. In this role, WRC assisted with a tremendous variety of organism cultures ranging from mammalian cell culture, fungi, bacteria, nematode and plant tissue culture. This broad array of research interests and experience is reflected in the diverse range of organisms cultured in this work.

## Supplementary Material

Additional file 1: Figure S1 Microbial collage. A collage of various microbial cultures photographed under consistent conditions (resuspended at OD_R_=2 on a photographic light bench, constant aperture and exposure). Cultures l.t.r: cyanobacteria (*Synechocystis* 6803), algae *Botryococcus braunii*, *Rhodobacter capsulatus* grown under three different conditions: autotrophic (H_2_, O_2_, CO_2_), photo-heterotrophic (anaerobic, succinic acid), heterotrophic (aerobic), *Rhodobacter sphaeroides* (anaerobic photoheterotrophic), *Ralsotonia eutropha* (R.e.), *E. coli* DH5α, *Candida molishiana* (yeast), *Chorella vulgaris* and *Chlamydomonas reinhardtii* both grown photosynthetically. Click here for file

Additional file 2: Figure S2 Scattering Reduction in Rhodobacter. Reduction of the scattering component of the ‘absorption spectrum’ of *Rhodobacter capsulatus* by resuspension in increasing levels of bovine serum albumin (BSA). *R. sphaeroides* was grown on YCC media under anaerobic photo-heterotrophic conditions which results in the induction of the light-harvesting proteins, with LH-2 being dominant (absorption peaks of 800-nm and 855-nm (Klug & Cohen, 1988). The light attenuation spectra were normalized at 860-nm. Click here for file

Additional file 3 **Recommended approach to quantifying pigments using OD measurements.** Combining OD measurements at a robust (predominantly scattering) and sensitive (absorption enhanced) wavelengths provides the best opportunity to correlate pigmentation levels within a microbial suspension. Click here for file

Additional file 4: Figure S4 **OD flow cell schematics.** Fabrication details for the OD flow cell including: **A)** Schematic and mechanical drawing of the prototype acrylic flow cell which includes an LED insert and photodiode, and **B)** The photo-diode used in this work showing the basic wiring arrangement to measure the output voltage which responds to the light intensity reaching the 2.29 mm x 2.29 mm photosensitive ‘window’ of the integrated circuit. Click here for file

Additional file 5: Figure S5 Algae OD measurements in OD flow cell. Ability of a short path-length flow cell to extend the range of OD measurements . *Chlorella vulgaris* cultures grown photosynthetically were resuspended at various cell concentrations and run through the flow cell as compared to undiluted OD_550_ measurements (Spectramax photometer). Click here for file
