# Supplementary material for: Improving accuracy of cell and chromophore concentration measurements using optical density
Source: BMC Biophys. 2013 Apr 22;6:4. doi: 10.1186/2046-1682-6-4 (PMC3663833; doi:10.1186/2046-1682-6-4)
Supplement: Additional file 1: Figure S1 — Microbial collage. A collage of various microbial cultures photographed under consistent conditions (resuspended at ODR=2 on a photographic light bench, constant aperture and exposure). Cultures l.t.r: cyanobacteria (Synechocystis 6803), algae Botryococcus braunii, Rhodobacter capsulatus grown under three different conditions: autotrophic (H2, O2, CO2), photo-heterotrophic (anaerobic, succinic acid), heterotrophic (aerobic), Rhodobacter sphaeroides (anaerobic photoheterotrophic), Ralsotonia eutropha (R.e.), E. coli DH5α, Candida molishiana (yeast), Chorella vulgaris and Chlamydomonas reinhardtii both grown photosynthetically. [file 2046-1682-6-4-S1.pptx]

## Slide 1
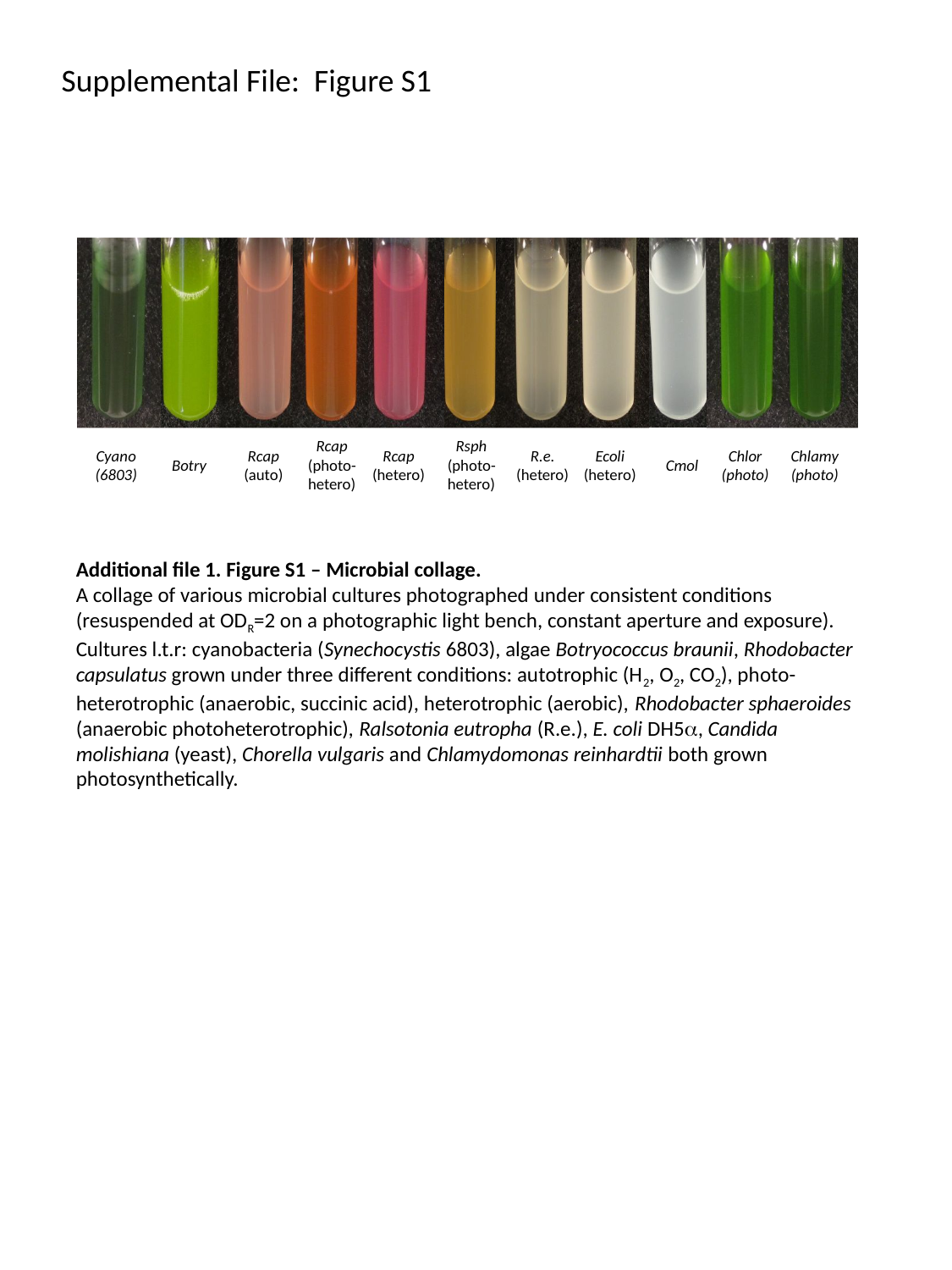

Supplemental File: Figure S1
Rcap
(photo-
hetero)
Rsph
(photo-
hetero)
Cyano
(6803)
Rcap
(auto)
Rcap
(hetero)
R.e.
(hetero)
Ecoli
(hetero)
Chlor
(photo)
Chlamy
(photo)
Botry
Cmol
Additional file 1. Figure S1 – Microbial collage.
A collage of various microbial cultures photographed under consistent conditions
(resuspended at ODR=2 on a photographic light bench, constant aperture and exposure).
Cultures l.t.r: cyanobacteria (Synechocystis 6803), algae Botryococcus braunii, Rhodobacter capsulatus grown under three different conditions: autotrophic (H2, O2, CO2), photo-heterotrophic (anaerobic, succinic acid), heterotrophic (aerobic), Rhodobacter sphaeroides (anaerobic photoheterotrophic), Ralsotonia eutropha (R.e.), E. coli DH5a, Candida molishiana (yeast), Chorella vulgaris and Chlamydomonas reinhardtii both grown photosynthetically.
