# Supplementary material for: Improving accuracy of cell and chromophore concentration measurements using optical density
Source: BMC Biophys. 2013 Apr 22;6:4. doi: 10.1186/2046-1682-6-4 (PMC3663833; doi:10.1186/2046-1682-6-4)
Supplement: Additional file 2: Figure S2 — Scattering Reduction in Rhodobacter. Reduction of the scattering component of the ‘absorption spectrum’ of Rhodobacter capsulatus by resuspension in increasing levels of bovine serum albumin (BSA). R. sphaeroides was grown on YCC media under anaerobic photo-heterotrophic conditions which results in the induction of the light-harvesting proteins, with LH-2 being dominant (absorption peaks of 800-nm and 855-nm (Klug & Cohen, 1988). The light attenuation spectra were normalized at 860-nm. [file 2046-1682-6-4-S2.pdf]

Supplemental File:  Figure S2

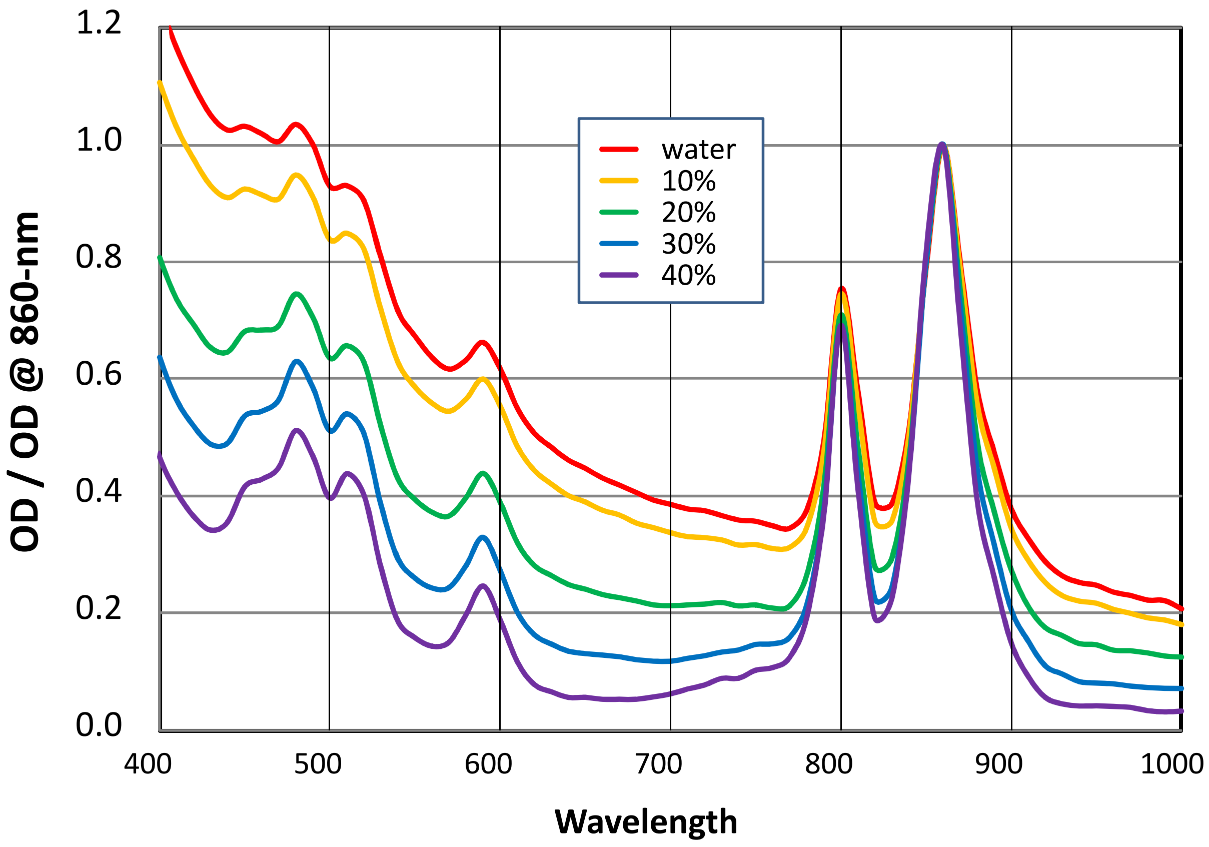


**Additional file 2. Figure S2 – Scattering Reduction in Rhodobacter.**
Reduction of the scattering component of the 'absorption spectrum' of *Rhodobacter capsulatus* by resuspension in increasing levels of bovine serum albumin (BSA).  *R. sphaeroides* was grown on YCC media under anaerobic photo-heterotrophic conditions which results in the induction of the light-harvesting proteins, with LH-2 being dominant (absorption peaks of 800-nm and 855-nm (Klug & Cohen, 1988).   The light attenuation spectra were normalized at 860-nm.
