# Supplementary material for: Improving accuracy of cell and chromophore concentration measurements using optical density
Source: BMC Biophys. 2013 Apr 22;6:4. doi: 10.1186/2046-1682-6-4 (PMC3663833; doi:10.1186/2046-1682-6-4)
Supplement: Additional file 3 — Recommended approach to quantifying pigments using OD measurements. Combining OD measurements at a robust (predominantly scattering) and sensitive (absorption enhanced) wavelengths provides the best opportunity to correlate pigmentation levels within a microbial suspension. [file 2046-1682-6-4-S3.pdf]

Additional File 3: Recommended approach to quantifying pigments using OD measurements.

An approach is presented for quantifying pigment in cells using the sensitive optical density of the absorption peak of the pigment ($OD_{S,+P}$) and at a robust scattering-dependent optical density at another wavelength (OD_R). The figure below illustrates the calculation carried out by measuring the absorption spectra of *E. coli* (which contains no pigment) in the presence of the dye trypan blue (an exclusion dye which remains outside living cells).

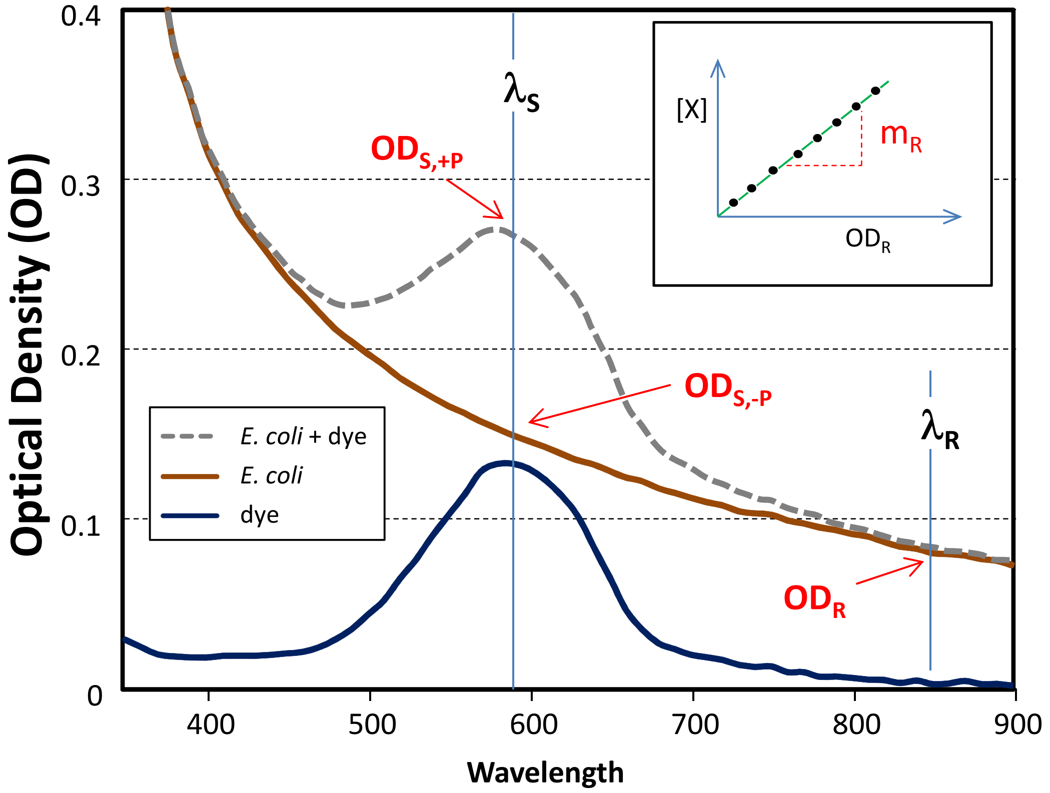

The concentration of dye will be proportional to the absorption beyond the scattering contribution of *E. coli* at the sensitive wavelength.

$$[P] = OD_{S,+P} - OD_{S,-P} \qquad \text{Eq. S3.1}$$

The scattering profile of *E. coli* allows calculation of the scattering contribution at the sensitive wavelength from the robust wavelength that is not effected buy the pigment. From Equation 3:

$$OD_{S,-P} = \delta \cdot OD_R \qquad \text{Eq. S3.2}$$

Finally, the correlation between cell concentration and the robust optical density stated in equation 1 (m_R=[X]/OD_R) provides the conversion factor needed to express the pigment levels per gram dry weight:

$$\frac{[P]}{[X]} = \frac{P}{X} = \frac{1}{m_R}\left(\frac{OD_{S,+P}}{OD_R} - \delta\right) \qquad \text{Eq. S3.3}$$

This approach was shown to allow for accurate prediction of the trypan blue dye concentration added to an *E. coli* culture (data not shown). The full utility of this approach for a pigmented organism will require estimation of the pigment-free optical density ($OD_{S,-P}$) which can be estimated from the

spectrum by extrapolating the scattering region (Fig. S3) or by experimentally measuring the pigment level for the correlation. It would be particularly useful to improve the accuracy of this approach by attenuating the scattering component as described in figures 6B and S2.
