# Supplementary material for: Improving accuracy of cell and chromophore concentration measurements using optical density
Source: BMC Biophys. 2013 Apr 22;6:4. doi: 10.1186/2046-1682-6-4 (PMC3663833; doi:10.1186/2046-1682-6-4)
Supplement: Additional file 4: Figure S4 — OD flow cell schematics. Fabrication details for the OD flow cell including: A) Schematic and mechanical drawing of the prototype acrylic flow cell which includes an LED insert and photodiode, and B) The photo-diode used in this work showing the basic wiring arrangement to measure the output voltage which responds to the light intensity reaching the 2.29 mm x 2.29 mm photosensitive ‘window’ of the integrated circuit. [file 2046-1682-6-4-S4.pdf]

Supplemental File: Figure S4

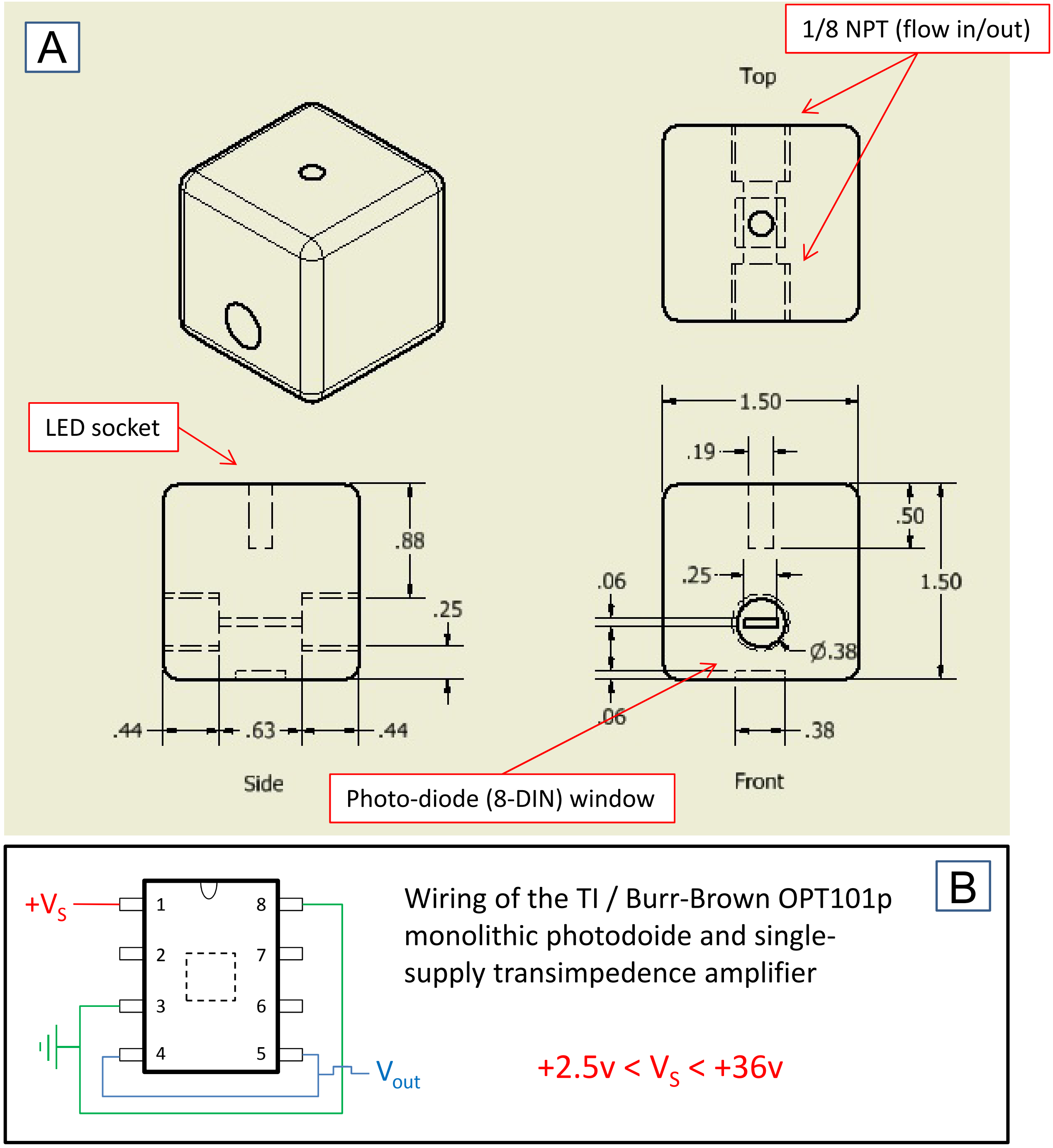

Additional file 4. Figure S4 – OD flow cell schematics.
Fabrication details for the OD flow cell including: **A)** Schematic and mechanical drawing of the prototype acrylic flow cell which includes an LED insert and photodiode, and **B)** The photo-diode used in this work showing the basic wiring arrangement to measure the output voltage which responds to the light intensity reaching the 2.29 x 2.29 mm photosensitive 'window' of the integrated circuit.
