# Supplementary material for: Improving accuracy of cell and chromophore concentration measurements using optical density
Source: BMC Biophys. 2013 Apr 22;6:4. doi: 10.1186/2046-1682-6-4 (PMC3663833; doi:10.1186/2046-1682-6-4)
Supplement: Additional file 5: Figure S5 — Algae OD measurements in OD flow cell. Ability of a short path-length flow cell to extend the range of OD measurements . Chlorella vulgaris cultures grown photosynthetically were resuspended at various cell concentrations and run through the flow cell as compared to undiluted OD550 measurements (Spectramax photometer). [file 2046-1682-6-4-S5.pdf]

Supplemental File: Figure S5

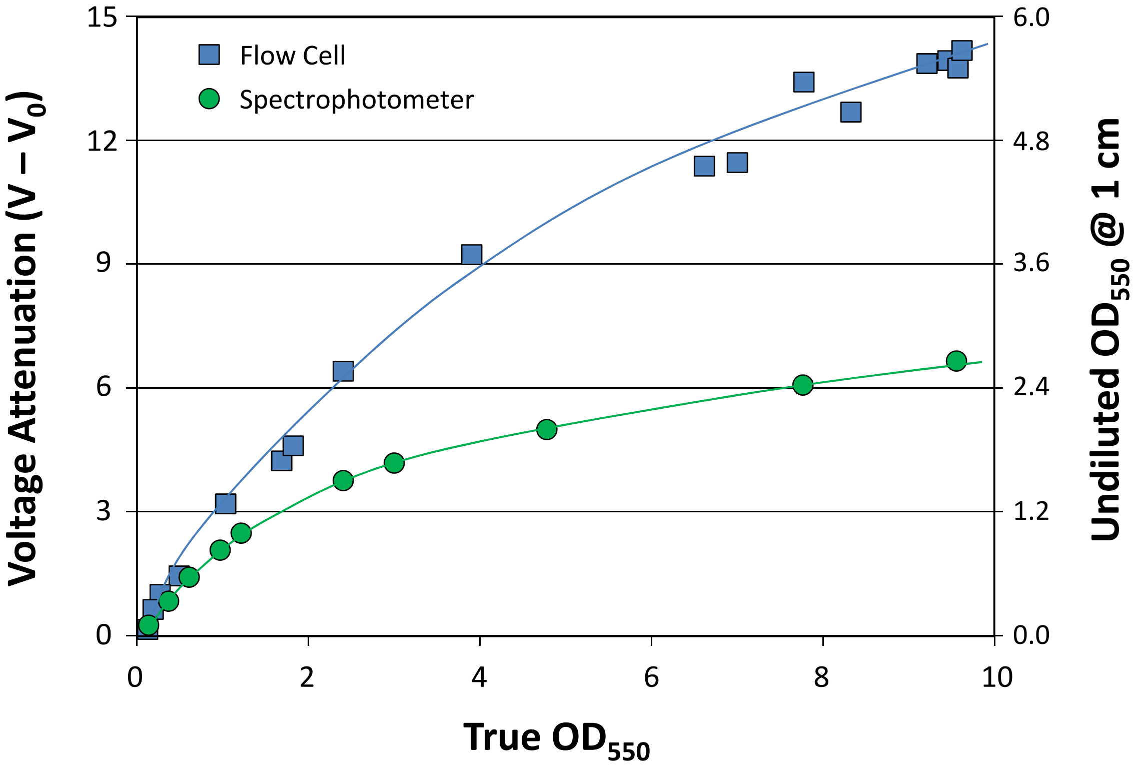

Additional file 5. Figure S5 – Algae OD measurements in OD flow cell.
Ability of a short path-length flow cell to extend the range of OD measurements . *Chlorella vulgaris* cultures grown photosynthetically were resuspended at various cell concentrations and run through the flow cell as compared to undiluted OD_{550} measurements (Spectramax photometer).
